# Nitrogen Oxide-Added Extracorporeal Membrane Oxygenation for Treating Critical Acute Heart Failure after Cardiac Surgery

**DOI:** 10.17691/stm2021.13.4.06

**Published:** 2021-08-28

**Authors:** V.V. Pichugin, S.E. Domnin, E.V. Sandalkin, S.A. Fedorov, V.V. Bober, S.A. Zhurko

**Affiliations:** Professor, Department of Anesthesiology, Resuscitation and Transfusiology; Privolzhsky Research Medical University, 10/1 Minin and Pozharsky Square, Nizhny Novgorod, 603005, Russia; Anesthesiologist-Resuscitator; Specialized Cardiosurgical Clinical Hospital named after Academician B.A. Korolev, 209 Vaneeva St., Nizhny Novgorod, 603136, Russia; Assistant, Department of Anesthesiology, Resuscitation and Transfusiology; Privolzhsky Research Medical University, 10/1 Minin and Pozharsky Square, Nizhny Novgorod, 603005, Russia; Cardiovascular Surgeon; Specialized Cardiosurgical Clinical Hospital named after Academician B.A. Korolev, 209 Vaneeva St., Nizhny Novgorod, 603136, Russia; Assistant, Department of Anesthesiology, Resuscitation and Transfusiology; Privolzhsky Research Medical University, 10/1 Minin and Pozharsky Square, Nizhny Novgorod, 603005, Russia; Cardiovascular Surgeon, Head of the Department of Cardiac Surgery; Specialized Cardiosurgical Clinical Hospital named after Academician B.A. Korolev, 209 Vaneeva St., Nizhny Novgorod, 603136, Russia

**Keywords:** critical acute heart failure, extracorporeal membrane oxygenation, nitric oxide

## Abstract

**The aim of the study** was to test the use of gaseous nitric oxide added to the extracorporeal membrane oxygenation (ECMO) system for treating critical acute heart failure after cardiac surgery.

**Materials and Methods:**

Venoarterial ECMO with addition of nitric oxide into the extracorporeal circuit was used for treating a 52-year-old patient after two-step cardiac surgery for acute dissection of the thoracic aorta and aortic valve insufficiency. After the Bentall–de Bono procedure, a technical error was revealed: that was a non-functioning anastomosis to the right coronary artery, which caused massive ischemic myocardial injury. An emergency myocardial revascularization was performed with the help of the cardiopulmonary bypass converted into ECMO; the patient’s condition improved and he was transferred to the ICU.

**Results:**

Considering the sharp decrease in heart contractility and the extremely high level of myocardial damage markers, it was decided to supply nitric oxide (40 ppm) to the ECMO circuit. A positive effect was noted within 8 h from the start of the procedure: the concentration of creatine phosphokinase-MB decreased almost 4 times and the concentration of troponin I decreased twofold. The most pronounced changes were observed by the end of day 1: a significant decrease in the concentration of myocardial damage markers, a decrease in the VIS indicator by 7.5 times; an improvement in the contractile function. Further on, the patient’s condition gradually stabilized: the manifestations of acute heart failure and multiple organ failure stopped, and then ECMO was discontinued after 82 h of work. The patient was decannulated and he continued to show stable hemodynamic parameters. He was discharged from the clinic on day 18 after surgery.

**Conclusion:**

For the first time, venoarterial ECMO with supply of gaseous nitric oxide into the extracorporeal circuit was used to support blood circulation after cardiac surgery. This made it possible to ensure the survival of the patient with critical ischemia-reperfusion injury developed after the surgery.

## Introduction

The most severe and rapidly progressing form of acute heart failure is the increasing instability of hemodynamics following the discontinuation of prolonged cardiopulmonary bypass. This clinical condition requires increasing doses of inotropic support to maintain the systolic blood pressure at >80 mm Hg and/or the cardiac index at >1.8 L/min/m^2^). Despite those measures, the hemodynamic parameters deteriorate, which often requires maximization of inotropic drug doses, and ultimately the use of mechanical support of blood circulation [1].

Using extracorporeal membrane oxygenation (ECMO) makes it possible to completely or partially replace the pumping function of the heart and/or the gas exchange function of the lungs to ensure the optimal level of blood circulation and metabolic processes in the patient’s body for a long time [2]. Nevertheless, a report from a leading Russian cardiac surgery center has shown that using ECMO after heart surgery is able to restore the adequate cardiac contractility function and gas exchange only in 36.6% of patients; according to this report, only 22% of patients were discharged from the clinic and the overall mortality rate was 78% [2].

To optimize the ECMO procedure, nitric oxide gas was proposed to be added into the extracorporeal circuit. In the available literature, only a few clinical studies addressed the effects of nitric oxide on extracorporeal circulation and patient’s hemodynamics [3]. Therefore, any new observation of this kind is of undoubted interest.

**The aim of the study** was to test the use of gaseous nitric oxide added to the ECMO system for treating critical acute heart failure after cardiac surgery.

## Materials and Methods

Patient B., 52 years old, was urgently admitted to the Specialized Cardiosurgical Clinical Hospital named after Academician B.A. Korolev (Nizhny Novgorod, Russia) where he was diagnosed with acute dissection of the thoracic aorta, aortic valve insufficiency, paroxysmal atrial fibrillation, cardiovascular failure IIA, and NYHA functional class III. The patient suddenly became ill 2 days before the admission; he felt a sharp piercing pain behind the sternum radiating into the inter-scapular area and further along the spine. The next day, a paroxysm of atrial fibrillation developed and was stopped with a medication (amiodarone 300 mg). The patient was admitted to the Uren District Hospital (Nizhny Novgorod Region, Russia), where echocardiography revealed an enlargement of the ascending aorta up to 7 cm with the presence of fluid in the pericardial cavity (about 200 ml). The patient was then transferred to the Specialized Cardiosurgical Clinical Hospital named after Academician B.A. Korolev for surgical treatment.

On admission: a serious condition, clear consciousness, an active position, skin and visible mucous membranes of a physiological color, normosthenic constitution. In the lungs: breathing with a bronchial component, no wheezing, respiratory rate — 18 per minute. Rhythmic heart sounds with a heart rate of 76 per minute. Auscultation — no heart sound abnormality. BP — 110/50 mm Hg under medication support. The abdomen is soft and painless. The liver is not enlarged. No peripheral edema. Physiological functions are normal.

ECG data on admission: sinus regular rhythm with a heart rate of 82 per minute, without signs of myocardial ischemia. Repeated echocardiography revealed an enlarged segment of the ascending aorta up to 70 mm, hemopericardium up to 200 mm.

Emergency surgery for vital signs was performed: a Bentall–de Bono procedure (conduit consisting of a MEDINZH-2 mechanical aortic valve with a diameter of 27 mm (MedInzh, Russia) and a Vascutek Gelweave vascular prosthesis (Vascutek Ltd., UK) with a diameter of 30 mm) and distal anastomosis area plastic using the sandwich technique. Normothermic perfusion was maintained for a total of 203 min. Crystalloid cardioplegia with Custodiol solution (4000 ml total) was used for myocardial protection. The aorta was clamped for a total of 164 min.

After the restoration of cardiac activity, signs of bleeding appeared; the source was found in the anastomosis to the right coronary artery (RCA). When trying to suture the anastomosis on the beating heart, the rate of bleeding increased. It was decided to extend this anastomosis by using an auto-venous graft. A fragment of the great saphenous vein 5 cm long was taken from the left tibia. The aorta was clamped again. The cardioplegic solution was administered into the root. The previous anastomosis to RCA and the conduit were removed. The RCA orifice was reduced to 5 mm and a distal anastomosis was formed of the auto-vein “end to end” along the axis. Then, a fragment of the auto-vein was replaced to the conduit and a proximal “end to side” anastomosis was applied along the axis directed to 6 o’clock. After the cardiac activity recovered, a pacemaker rhythm was imposed with a heart rate of 80 per minute. Hemodynamics got stabilized. Osteosynthesis of the sternum was performed using wire sutures. Surgical wounds on the chest and right groin were sutured layer-by-layer.

After the patient was transferred to the ICU, he experienced progression of acute heart failure, extreme instability of hemodynamics, and hyperenzymemia, which necessitated an increase in cardiotonic support. Since the patient’s heart rhythm was driven with a pacemaker, no ECG record for signs of ischemia was possible. A decision was made to perform an emergency selective coronary angiography: the test showed no filling of the RCA orifice; all subsequent attempts to conduct a coronary guidewire were unsuccessful. Diagnoses of acute coronary syndrome, acute lower Q-wave myocardial infarction, and condition after a Bentall–de Bono procedure were then made.

The patient underwent emergency myocardial revascularization by repeating RCA bypass grafting. Peripheral arteriovenous ECMO was initiated. Normothermic perfusion was run for a total of 60 min, while the aorta was clamped for 36 min. Revision of coronary arteries revealed no pulsation of RCA in plastic zone and the following additional auto-venous graft; both vessels looked collapsed.

The decision was made to bypass the posterior descending artery accessible to bypassing in its proximal third. A distal “end to side” anastomosis was performed between the posterior descending artery and the auto-vein along the axis, by using a 7-0 prolene without additional sutures. The shunt was connected to the vascular prosthesis. A proximal anastomosis of a venous shunt was formed with an orientation of 5 o’clock. As a result, the shunt showed pulsation and was well drained off. Cardiac activity in the form of ventricular fibrillation and asystole was recorded, a pacemaker was imposed. An attempt was made to slow down the heart-lung machine, which resulted in a decrease in the central hemodynamics indices. It was then decided to connect another ECMO apparatus via peripheral circulation. Thus, the femoral vein was exposed 2 cm below the inguinal fold on the right and cannulated with a 27 Fr cannula according to Seldinger. The ECMO procedure was launched by using a Stockert S3 perfusion system (Sorin Group, Germany) and a Medos HILITE 7000 oxygenator (Medos, Germany). With the help of this additional ECMO, the hemodynamics got stabilized. Decannulation of the right heart was performed, and the wound closed.

**Figure 1:**
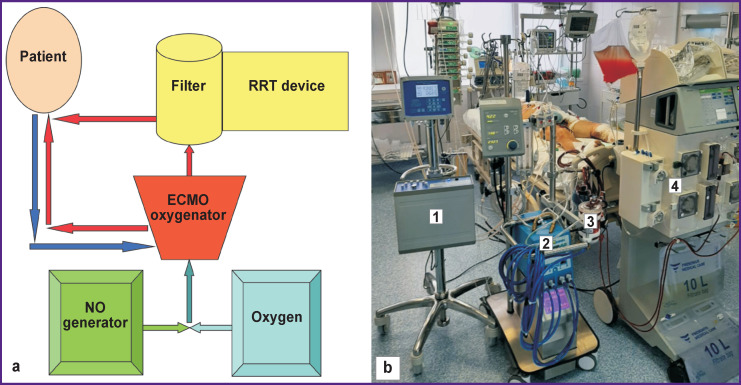
Supply of nitric oxide to the ECMO circuit in combination with a renal replacement therapy (RRT) session: (a) diagram; (b) photo: *1* — NO generator; *2* — ECMO setup; *3* — ECMO oxygenator; *4* — RRT device

The patient was transferred to the ICU in critical condition. Arteriovenous ECMO was performed at the following parameters: perfusion flow — 5.21 L/min; perfusion index — 2.4 L/min/m^2^). Using epinephrine and norepinephrine (VIS — 35 units) for inotropic support, we observed stable hemodynamic parameters: BP of 90–95/70–75 mm Hg. Ultrasound examination showed a sharp decrease in the left ventricular ejection fraction (LVEF) to 30%, the right ventricle — to 22% with diffuse hypokinesis. The myocardial damage markers were creatine phosphokinase-MB (CPK-MB) at 500 ng/ml, troponin I at 200 ng/ml. Hyperenzymemia was also noted: the level of AST increased up to 2331 units, ALT — to 972 units, CPK — to 6145 units, lactate dehydrogenase — to 2633 units. It was then decided to add gaseous nitric oxide to the ECMO circuit (Figure 1). To generate nitric oxide, we used a device (Tianox; RFNC-VNIIEF, Russia) that produced nitric oxide from the ambient air, supplied it into the extracorporeal circuit, and monitoring/regulating NO concentration in the supplying mixture [4, 5]. The average dosage of nitric oxide was 40 ppm; the average NO_2_ concentration was 0.2–1.1 (0.80±0.06) ppm. The level of methemoglobin (MetHb) at all stages of the procedure did not exceed 1.5%. A session of efferent therapy — prolonged hemodiafiltration (CVVHDF) was started after 3 h.

**Figure 2:**
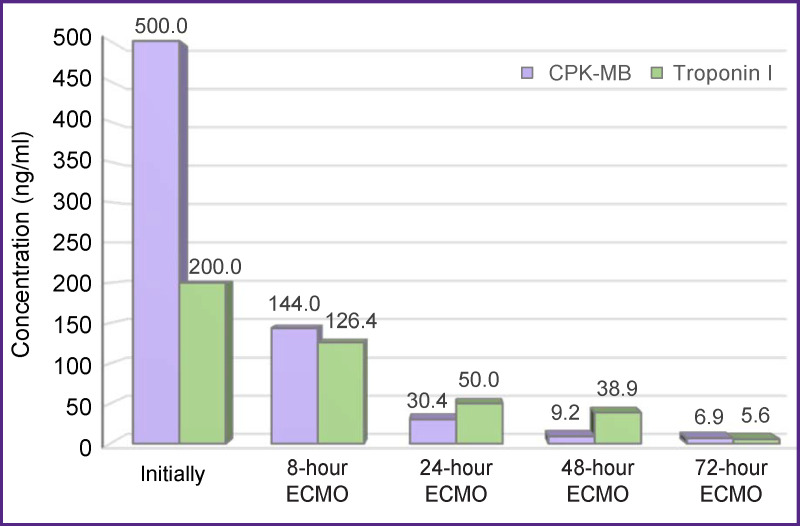
Myocardial damage markers in the patient’s blood during NO-containing ECMO

**Figure 3:**
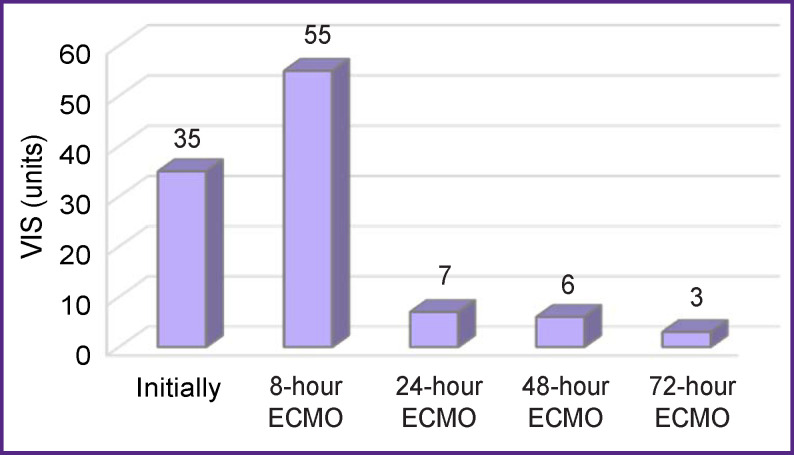
Changes in the VIS during NO-containing ECMO

After 8 h, a decrease in myocardial damage markers was noted: CPK-MB — down to 144 ng/ml, troponin I — down to 126 ng/ml (Figure 2). During that time, ECMO was performed with the calculating flow, and stable hemodynamics was maintained by increased doses of epinephrine and norepinephrine (VIS was 55 units — Figure 3). Diffuse hypokinesis of both left and right ventricles was noted and the LVEF decreased to 19%.

A significant improvement in the patient’s condition was observed only after 24 h exposure to NO-containing ECMO and ongoing hemodiafiltration. There was a decrease in both the dose of the inotropic drugs administered (VIS — to 7 units) and the concentration of the myocardial damage markers (CPK-MB — to 30.4 ng/ml, troponin I — to 50 ng/ml). The first hemodiafiltration session was completed after 24 h.

Fifty-eight hours after starting the procedure, a gradual decrease in ECMO performance was initiated by reducing the perfusion flow (75–50–25% of the total volume rate); during that period, nitric oxide was continuously supplied to the oxygenator. At this stage, an increase in LVEF up to 45% and restoration of contractility of both the left and right ventricles was noted. Myocardial damage markers also decreased: CPK-MB — to 9.2 and 6.9 ng/ml after 48 and 72 h, respectively; troponin I dropped to 38.9 and 5.6 ng/ml after 48 and 72 h, respectively. After 48 h, the VIS was reduced to 6 units, and after 72 h — to 3 units. Twelve hours after the first session, a second portion of renal replacement therapy (RRT) was performed to treat multiple organ failure. During the RRT session, the manifestations of acute heart failure and multiple organ failure almost completely ceased. ECMO was discontinued 82 h later and the patient was decannulated. He showed stable hemodynamic parameters and myocardial contractile function. On day 5 after the operation, the patient was extubated; he maintained clear consciousness, adequate spontaneous breathing, satisfactory acid-base balance, and close to normal parameters of the blood chemistry. In total, he stayed in the ICU for 11 days. The postoperative wound of the sternum healed by primary intention.

The patient was discharged from the clinic on day 18 after the operation. At the time of discharge, he had: ECG — with the sinus rhythm and a heart rate of 84 per minute; QRST — with no change; echocardiography — end-diastolic/systolic volume (EDV/ESV) — 161/83 ml; LVEF — 51%; the aortic valve prosthesis function was not impaired; aortic pressure gradient — 9/6 mm Hg; 0–I degree regurgitation; the mean pulmonary artery pressure was 32 mm Hg; 6-minute walk test — 280 m; Rehabilitation Routing Scale score was 4.

The work was performed in accordance with the Declaration of Helsinki (2013) and approved by the Ethics Committee of the Specialized Cardiosurgical Clinical Hospital named after Academician B.A. Korolev. The patient signed an informed consent to participate in the study.

## Results and Discussion

Extracorporeal membrane oxygenation is a standard therapy used in a variety of life-threatening clinical situations, such as acute respiratory failure, resuscitation after cardiac and respiratory arrest, cardiogenic shock, sepsis, circulatory support after cardiac surgery, and respiratory or heart transplants. Many of these situations (especially in venous-arterial ECMO) are associated with systemic inflammatory response syndrome (SIRS) or reperfusion injury after ischemia.

Studies in animals and humans demonstrated a positive effect of exogenous nitric oxide on the platelet function and minimization of SIRS after NO was mixed with the blood in the extracorporeal circuit [6]. It is also important that NO donors or gaseous nitric oxide are able to reduce ischemia-reperfusion injury in various organs, including the brain [7] and heart [8]. Thus, a randomized study by James et al. [9] showed that the addition of 20 ppm nitric oxide to the oxygenator gas supply during cardiopulmonary bypass in children undergoing surgery for congenital heart diseases, reduced the incidence of postoperative low cardiac output syndrome. An earlier study by Checchia et al. [10] also noted a decrease in the inflammatory response and the need for mechanical ventilation in children with tetralogy of Fallot operated under cardiopulmonary bypass.

A group from Australia [11] suggested that the addition of NO to the gas mixture in the oxygenator, during the entire ECMO procedure could limit ischemic and reperfusion injury to the heart, and also suppress the contact activation of platelets and leukocytes. The authors supplied 20 ppm NO to the oxygenator circuit along with other gases (air/oxygen) from the start of the ECMO procedure. The study included 30 children (31 procedures were performed, one child received two ECMO procedures). In 11 (35%) children, the reason for starting ECMO was cardiac arrest, in 10 (32%) — ineffectiveness of maximal drug therapy, and 6 (19%) patients could not be possibility to stop cardiopulmonary bypass. The mean peak level of MetHb was 1.2 (0.4–2.6%); no child showed the need to stop NO or inject methylene blue to curb MetHb. Eight children (26%) required hemofiltration during the ECMO, but only one of them died. In total, 25 children (83%) survived to be discharged from the hospital; five died (17%). The results of the conventional ECMO performed by the authors in 101 patients during three years were as follows: out of 101 children, 33 patients died, i.e. the mortality rate was 32.7%.

The present clinical case is the first successful example of using NO-containing venoarterial ECMO in the Russian Federation. It should be noted that at present, the systems for delivering nitric oxide into the extracorporeal circuit are quite complex and cumbersome; in contrast, the Tianox nitric oxide generator we used, made it possible to overcome these technical problems.

The indication for starting ECMO in this patient was a surgical mishap during the first operation, which caused massive myocardial ischemic injury. The selected treatment — urgent myocardial revascularization and conversion of the cardiopulmonary bypass into ECMO — made it possible to stabilize the patient’s condition and transfer him to the ICU. Considering the sharp decrease in the contractile function and the extremely high levels of myocardial markers, we decided to supply nitric oxide to the extracorporeal ECMO circuit. The positive effect of the procedure was noted within 8 h from its beginning: the concentration of CPK-MB decreased almost 4-fold and the concentration of troponin   I decreased twofold. At the same time, no significant improvement in myocardial contractility was observed: the VIS index increased to 55 units, the LVEF decreased to 19%. Undoubtedly, the ultrahemodiafiltration session also contributed to the successful treatment.

The most pronounced changes appeared by the end of the first day: a significant decrease in the concentration of myocardial markers, a decrease in the VIS indicator by 7.5 times, an improvement in the indicators of myocardial contractility. It was at this stage that the patient’s condition was stabilized. Later on, his condition was characterized by consistent stabilization: the signs of acute heart failure and multiple organ failure did not appear anymore, and then ECMO was discontinued (total duration — 82 h). The patient was decannulated and he continued to show stable hemodynamic parameters. He was discharged from the clinic on day 18 after surgery with satisfactory indicators of myocardial contractility.

This clinical case has shown that the administration of NO into the gas mixture of ECMO is safe and does not cause side effects. No severe methemoglobinemia or bleeding was observed. However, additional research is needed to improve the survival of patients with acute myocardial damage.

## Conclusion

For the first time, venoarterial ECMO with supply of gaseous nitric oxide into the extracorporeal circuit was used to support blood circulation after cardiac surgery. This made it possible to ensure the survival of a patient with critical ischemia-reperfusion injury of the myocardium after surgery.

## References

[1] Bautin A.E., Mikhaylov A.P., Laletin D.A., Rubinchik V.Ye. (2014). Right ventricle contractility during early postoperative period after coronary artery bypass grafting with cardiopulmonary bypass.. Patologiya krovoobrashcheniya i kardiokhirurgiya.

[2] Bockeria L.A., Shatalov K.V., Makhalin M.V (2017). The use of extracorporeal membrane oxygenation in adult cardiac surgery in a clinic in the development of cardiac or respiratory failure in the early postoperative period.. Kliniceskaa i eksperimentalʹnaa hirurgia. Zurnal imeni akademika B.V. Petrovskogo.

[3] Pichugin V.V., Seyfetdinov I.R., Medvedev A.P., Domnin S.E (2019). Inhaled nitric oxide in the prevention of ischemic and reperfusion injuries of the heart during operations with cardiopulmonary bypass.. Medicinskij alʹmanah.

[4] Karelin V.I., Buranov S.N., Pimenov O.A., Selemir V.D., Shirshin A.S (2013). Plasma-chemical facility for NO-therapy.. Medial’.

[5] Buranov S.N., Buyanov A.B., Voevodin S.V., Karelin V.I., Selemir V.D., Shirshin A.S (2016). Apparatus for inhalation NO-therapy.. Bioradikaly i antioksidanty.

[6] Skrzypchak A.M., Lafayette N.G., Bartlett R.H., Zhou Z., Frost M.C., Meyerhoff M.E., Reynolds M.M., Annich G.M (2007). Effect of varying nitric oxide release to prevent platelet consumption and preserve platelet function in an in vivo model of extracorporeal circulation.. Perfusion.

[7] Kida K., Shirozu K., Yu B., Mandeville J.B., Bloch K.D., Ichinose F (2014). Beneficial effects of nitric oxide on outcomes after cardiac arrest and cardiopulmonary resuscitation in hypothermia treated mice.. Anaesthesiology.

[8] Heusch G (2015). Molecular basis of cardioprotection: signal transduction in ischemic pre-, post-, and remote conditioning.. Circ Res.

[9] James C., Millar J., Horton S., Brizard C., Molesworth C., Butt W (2016). Nitric oxide administration during paediatric cardiopulmonary bypass: a randomised controlled trial.. Intensive Care Med.

[10] Checchia P.A., Bronicki R.A., Muenzer J.T., Dixon D., Raithel S., Gandhi S.K., Huddleston C.B (2013). Nitric oxide delivery during cardiopulmonary bypass reduces postoperative morbidity in children — a randomized trial.. J Thorac Cardiovasc Surg.

[11] Chiletti R., Horton S., Bednarz A., Bartlett R., Butt W (2018). Safety of nitric oxide added to the ECMO circuit: a pilot study in children.. Perfusion.

